# Primary bovine skeletal muscle cells enters apoptosis rapidly via the intrinsic pathway when available oxygen is removed

**DOI:** 10.1371/journal.pone.0182928

**Published:** 2017-08-08

**Authors:** Sissel Beate Rønning, Petter Vejle Andersen, Mona Elisabeth Pedersen, Kristin Hollung

**Affiliations:** Nofima AS, Ås, Norway; International Nutrition Inc, UNITED STATES

## Abstract

Muscle cells undergo changes post-mortem during the process of converting muscle into meat, and this complex process is far from revealed. Recent reports have suggested programmed cell death (apoptosis) to be important in the very early period of converting muscle into meat. The dynamic balance that occurs between anti-apoptotic members, such as Bcl-2, and pro-apoptotic members (Bid, Bim) helps determine whether the cell initiates apoptosis. In this study, we used primary bovine skeletal muscle cells, cultured in monolayers *in vitro*, to investigate if apoptosis is induced when oxygen is removed from the growth medium. Primary bovine muscle cells were differentiated to form myotubes, and anoxia was induced for 6h. The anoxic conditions significantly increased (P<0.05) the relative gene expression of anti- and pro-apoptotic markers (Aif, Bcl-2, Bid and Bim), and the PARK7 (P<0.05) and Grp75 (Hsp70) protein expressions were transiently increased. The anoxic conditions also led to a loss of mitochondrial membrane potential, which is an early apoptotic event, as well as cytochrome c release from the mitochondria. Finally, reorganization and degradation of cytoskeletal filaments occurred. These results suggest that muscle cells enters apoptosis via the intrinsic pathway rapidly when available oxygen in the muscle diminishes post-mortem.

## Introduction

A major quality trait in beef is meat tenderness; however, the molecular mechanisms of the tenderisation process remains to be fully determined. The meat tenderisation process is enzymatic, involving several intracellular proteolytic systems that degrade cell constituents, including caspases, calpains, proteasome, cathepsins and matrix metalloproteinases [1]. Skeletal muscles empty oxygen levels soon after slaughter, inducing cell death. Traditionally, this has been considered as necrosis, however, no inflammation is observed [2]. After animal bleeding, muscle enter an anoxic state which affects all metabolic pathways, and muscle cells eventually enter cell death/survival pathways [1]. The natural and targeted cause of cell death, namely programmed cell death (apoptosis), is shown to be important in the very early period of converting muscle into meat (reviewed in [1–4]). Recent reports show that the intrinsic pathway, involving caspase 3 activation and cytochrome c release from mitochondria to the cytoplasm, is the main apoptosis pathway during post- mortem meat tenderization [5].

Apoptosis is important during normal development and aging, and to maintain cell populations in tissue. It is also an important defence mechanism during immune reactions and exposure to cytotoxic compounds, heat, radiation, hypoxia (loss of oxygen) or infections (reviewed in [6]). Apoptosis can be distinguished from necrosis (traumatic cell injury that is fatal to the cell) by its unique morphological and biochemical alterations. Apoptosis is triggered by external (death receptor pathway) or internal stimuli (mitochondrial pathway). In the intrinsic pathway, the cells undergo apoptosis because of cell stress, while the extrinsic pathway is induced by signals from other cells. Both pathways initiates apoptosis by activating caspases. It is an energy-dependent cascade of molecular events that eventually leads to termination, including activation of caspases, DNA fragmentation, degradation of cytoskeletal and nuclear proteins, formation of apoptotic bodies and finally uptake by phagocytic cells [6]. Cells can also die while displaying a morphology similar to apoptosis without caspase activation. This is called caspase-independent apoptosis, and is linked to the release of apoptosis inducing factor (AIF) from the mitochondria into the nucleus [7]. The morphological characteristics of apoptosis are consistent in most cell types; however, apoptosis in skeletal muscle is still relatively unexplored. Both caspase-dependent as well as -independent pathways have been implicated in muscle. Siu *et al*., for example demonstrated increased caspase-3 activity and release of cytochrome c, as well as increased AIF expression in differentiated myotubes during H_2_O_2_-mediated oxidative stress [8]. Likewise Lie *et al*., found that caspase-3 activation did not increase during muscle atrophy although apoptosis increased accordingly [9]. Skeletal muscles fibres are multinucleated and the mitochondrial content differs between fibre types, and these factors add complexity to apoptotic mechanisms in skeletal muscle.

Apoptosis is believed to be the first step in the conversion of muscle into meat [10], but the molecular mechanism in this process is still not fully characterized. Understanding the biochemical processes after slaughter may help us provide better solutions for post-slaughter interventions to improve meat quality. We used primary bovine skeletal muscle cells to investigate this further *in vitro*, mimicking the slaughter condition by removing oxygen (anoxia).

## Materials and methods

### Antibodies

Immunofluorescence: Mouse anti-cytochrome c (1:100 dilution, ab65311) and rabbit anti-desmin (1:80 dilution, ab8592) were from Abcam (Cambridge, UK). Mouse anti-α-tubulin (1:2000 dilution, T5168) was from Sigma Aldrich (St.Lois, MO, US). Alexa 488 goat anti-mouse, Alexa 546 goat anti-mouse, and Alexa 488 goat anti-rabbit were from Invitrogen (Carlsbad, CA, USA). Hoechst and Alexa-488 Phalloidin was from Molecular probes (Invitrogen, Paisley, UK). Western blot: Mouse anti-Grp75 (Hsp70) (ab13529, 1:2000 dilution), rabbit anti-PARK7 (ab37180, 1:2000 dilution), rabbit anti-actin (1:500 dilution, ab1801), rabbit anti-desmin (1:2000 dilution, ab8592) were from Abcam. Mouse anti-α-tubulin (1:10 000 dilution, T5168) was from Sigma Aldrich (St. Lois, MO, US). CY3-conjugated goat anti-mouse and CY5-conjugated goat anti-rabbit (1:2500 dilution, PA43010 and PA45011, respectively), were procured from GE Healthcare (Buckinghamshire, UK). JC-1 Mitochondrial membrane potential probe was from Thermo Fisher Scientific (Waltham, MA, US) and Z-LEHD-FMK (irreversible caspase-9 inhibitor, 20 μM) was from Abcam (Cambridge, UK)).

### Bovine primary skeletal muscle cell isolation

Although *in vitro* cell models can never replace the use of *in vivo* animal models, the cell models can provide valuable knowledge including endogenous effect, dose-response and target organ, at low cost and short time compared to using *in vivo* animal models. In addition, by using cell models, we can test one specific condition at a time, and are thus able to say that any response noted is most likely due to that condition. We have previously developed a model system using bovine primary skeletal muscle cells [11]. The bovine primary skeletal muscle cells were obtained from a collection of animals of the same age (young animals), gender and breed using hot boned fresh muscle samples obtained immediately after slaughter from *Longissimus thoracis* (beef sirloin) collected at an industrial abattoir (Nortura AS, Rudshøgda, Norway). The isolated cells were cultured, transferred into 75 cm^2^ coated culture flasks, and then stored in DMSO in liquid nitrogen until further use. We performed all experiments in the second or third cell passage, and on at least three independent cell seedings.

### Cell culture and treatment

Tissue culture coverslips (Menzel-Gläser, Braunschweig, Germany), 96, 6- and 24-well plates (BD Falcon, Franklin Lakes, NJ, USA), or cell culture flasks (VWR, West Chester, PA, USA) were coated with 3 μl/cm^2^ Entactin-Collagen IV-Laminin (1 mg/ml, Millipore, Billerica, MA, USA). Subsequently the coated surface was washed twice with PBS before culturing the cells. The primary cells were grown in Dulbecco’s modified Eagles’s medium (DMEM) with L-glutamine (2 mM), 2% FBS, 2% Ultroser G, P/S (10 000 units/ml), and Amphotericin B (250 μg/ml) until 70–80% confluence (usually 3 days). The cells were then washed with PBS and placed in differentiation medium (DMEM, 2% FBS, P/S, Amphotericin B, and 25 pmol insulin) to induce myogenesis. Oxygen scavenging was performed on differentiated muscle cells (3 days of differentiation).

### Oxygen scavenging/anoxia culturing conditions

We used an EC-Oxyrase obtained from Oxyrase, Inc. (Mansfield, OH, USA) to scavenge O_2_ from the medium. EC-Oxyrase is an enzyme system that depletes the medium of oxygen when a substrate, such as sodium lactate, is available. In this method, O_2_ was scavenged from the incubation medium by adding sodium lactate (9.9%) and the oxygen scavenger EC-Oxyrase (1%) for the indicated time periods. Percentage O_2_ saturation was measured using an oxygen probe (Presens, Regentsburg, Germany) (S1 Fig). This method has been employed successfully by several researchers to produce hypoxic/anoxic conditions to examine cellular responses in a variety of cell cultures [12–14].

### RNA isolation and real-time PCR

Cell cultures treated as indicated in the figure legends were washed twice with PBS and purified by RNeasy mini kit including a DNase treatment according to the manufacturer’s protocol (Qiagen, Hilden, Germany). cDNA was generated from ~200 ng mRNA using TaqMan® Reverse Transcription Reagents (Invitrogen, Carlsbad, CA, USA) according to the manufacturer’s protocol. The cDNA was diluted four times before aliquots (in triplicates) were subjected to real-time PCR analysis using an ABI Prism 7700 Sequence Detection system (Applied Biosystem, UK). The real-time PCR reaction volume of 25 μl contained 4 μl template cDNA, 0.2 μM of each primer, 0.1 μM probe, 1.25 units Taq DNA polymerase (AmplitaqGold, Applied Biosystems, Carlsbad, CA, USA), 0.3 units uracil N-glycosylase (AmpErase UNG, Applied Biosystems), 0.2 mM dATP, dCTP, dGTP and 0.4 mM dUTP (Applied Biosystems), 5 mM MgCl2, and 1 x TBA buffer. The cycling profile was as follows: An initial decontamination step for 2 min at 50°C to allow optimal UNG enzymatic activity, followed by a denaturation step of 10 min at 95°C, followed by 40 repeats of 15 s denaturation at 95°C and 60 s synthesis at 60°C. A list of primers and probes used is provided in Table 1. Primers and probes were designed using the Primer Express Program (Applied Biosystems). Gene expression in the samples was normalized against TATA and EF1, and ΔCt was calculated, according to the MIQE guidelines [15]. The results using TATA and EF1 were similar, therefore only TATA was chosen for further analyses. PCR efficiency and melting point analysis were performed on all targets. Comparison of the relative gene expression between control and treated cells was derived by using the comparative Ct method. In short, values were generated by subtracting ΔCt values between two samples, which gives a ΔΔCt value. The relative gene expression was then calculated by the formula 2^-ΔΔCt^. The efficiency of each set of primers was always higher than 96%. The real-time PCR was performed in technical triplicates on at least three independent experiments seeded out in duplicates.

**Table 1:** List of primers and probes used for quantitative real-time PCR.

Primers and probes	Sequence	GeneBank accession. no
*Reference genes*:		
Ef1	Fwd-5’-CCTGGCAAGCCCATGTGT-3’	XR_083736
	Rev-5’-TGTCACGCACAGCAAAACG-3’	
	P-5’-CGAGAGCTTCTCTGATTATCCTCCCCTGG-3’	
TATA	Fwd-5’-CGTTTTGCTGCTGTAATCATGAG -3’	NM_001075742
	Rev-5’-CCATCTTCCCAGAACTGAATATCA-3’	
	P-5’-ATAAGAGAGCCCCGCACCACTGCA-3’	
*Pro-and anti-apoptotic markers*		
Aif	Fwd-5’-GATCCTGATGTATGAAGAGAAAG-3’CAA	XM_015459866.1
	Rev-5’-AATCAGGGCAACTCAGAGATAGCT-3’	
	P-5’-AGAAGCCAACAGGTCTCCCAGCCAA-3’	
Bax	Fwd-5’-TTTCTGACGGCAACTTCAACTG-3’	XM_015458140.1
	Rev-5’-GGTGCACAGGGCCTTGAG-3’	
	P-5’-TTGTCGCCCTTTTCTACTTTGCCAGCA-3’	
Bcl2	Fwd-5’-GGAGCTGTATGGCCCTAGCA-3’	NM_001166486.1
	Rev-5’-TGAGCAGTGCCTTCAGAGACA	
	P-5’-CGGCCCCTGTTTGATTTCTCCTGG-3’	
Bid	Fwd-5’-GCTTCGGCCACTGATCCA	NM_001075446.2
	Rev-5’-CCCCGGGCTTTAAAATGGT	
	P-5’-CCCAAGACGATCACGGAGTGCCA-3’	
Bim	Fwd-5’-GCCCGGCACCCATGA-3’	XM_010809718.2
	Rev-5’-TTGAAGGCCTGGCAAGGA-3’	
	P-5’-TGTGACAAATCCACACAGACCCCAAGC-3’	

### Western blotting

Cell cultures treated as indicated in figure legends were washed twice with PBS, before addition of lysis buffer (10 mM Tris, pH 6.8, 5 mM EDTA, 50 mM NaF, 30 mM sodium pyrophosphate, 2% (w/v) sodium dodecyl sulphate (SDS), containing AEBSF and phosphatase cocktail inhibitor II). Cell debris was removed by centrifugation at 13 000 x g for 10 min at 4°C, and the cleared lysate was subjected to SDS-Page gel electrophoresis. Following electrophoresis, the proteins were transferred onto nitrocellulose membranes using an iBlot Gel Transfer Device (Invitrogen, Carlsbad, CA, USA)). All membranes were blocked with 2% ECL Advanced blocking agent (GE Healthcare) in Tris buffered saline (TBS)-tween for 1 h at RT. Primary and secondary antibodies were diluted in 0.5% blocking agent and incubated for 1.5 h at RT (or ON at 4°C) with gentle shaking. Membranes were washed 3 x 10 min with TBS-tween after both incubations. Blots were scanned and visualized using Ettan DIGE Imager (GE Healthcare), and the images were analysed and quantified using ImageQuant TL software (GE Healthcare).

### Immunocytochemistry and fluorescence microscopy

Differentiated muscle cells were grown on coated coverslips (Assistent, Sondheim/Rhön, Germany), washed in PBS and fixed in either 2% PFA (Reidel-de Haën, Seelze, Germany) or ice-cold ethanol for 15 min. The cells were washed three times in PBS, permeabilized using 0.1% Triton X-100 in PBS and incubated with 5% non-fat dry milk for 30 min before incubation with primary antibody for 1 h. Subsequent incubation with secondary antibodies was performed for 30 min before using Dako fluorescent mounting medium (Glostrup, Denmark). The cells were examined either by confocal microscopy (Leica TCS SP5, Mannheim, Germany) or by fluorescence microscopy analysis (apotome mode) (ZEISS Axio Observer Z1 microscope, Jena, Germany), and images were processed using Adobe Photoshop CS3. Brightness and contrast, if used, were adjusted manually across the entire image. The objective used by confocal microscopy (Leica) was HCX PL APO 1.25 oil, while the objective used with fluorescence microscopy was a LCI Plan-Neofluor 25x/ 0.8 1mm Korr M277 objective oil.

### Measurement of mitochondrial membrane potential

We used a lipophilic cationic dye (JC-1) to measure mitochondrial membrane potential. This dye selectively enters the mitochondria, and changes the colour from red to green as the membrane potential decreases. JC-1 spontaneously forms aggregates with intense red fluorescence in healthy cells, while in apoptotic cells JC-1 will remain in a monomeric form, showing only green fluorescence.

### Cell viability, live/dead viability/cytotoxicity kit and caspase assays

Cells grown on coated 96-well multiwall plates were washed in PBS before analysis. Cell viability was measured as the number of viable cells in culture based on quantitation of the ATP present, which signals the presence of metabolically active cells, and was performed according to the protocol (CellTiter-Glo® Luminescent Cell Viability Assay, Promega, WI, USA). Adding CellTiter-Glo® to cell cultures results in cell lysis and generation of a luminescent signal proportional to the amount of ATP present. According to the manufacturer, the amount of ATP is shown to be directly proportional to the number of cells present in culture. To quantify apoptotic cell death we used Live/dead viability/cytotoxicity kit (Molecular probes, Invitrogen, Paisley, UK). This kit is based on the simultaneous determination of live and dead cells with two probes that recognises cell viability. In live cells, the nonfluorescent calcein AM, is converted to a green-fluorescent calcein after acetoxymethyl ester hydrolysis by intracellular esterases. The cell-permeant viability-indicator ethidium homodimer-1 (EthD-1) is a high-affinity nucleic acid stain that is weakly fluorescent until bound to DNA and emits red fluorescence. When cells die, the plasma membranes of those cells become disrupted and ethidium homodimer then enter the cells and bind to DNA. Live cells have intact membrane, and therefore the ethidium homodimer cannot enter. Caspase 3/7 and 9 activities were measured according to the protocol (Caspase-Glo® 3/7 Assay and Caspase Glo® 9 Assay, Promega, WI, USA).

### Statistical analysis

Significant variance by treatments in comparison to the untreated sample was determined by two-way ANOVA performed in GraphPad Prism version 6.0 (GraphPadSoftware, La Jolla, CA, USA). Differences were considered significant at p≤0.05.

## Results and discussion

**Fig 1:**
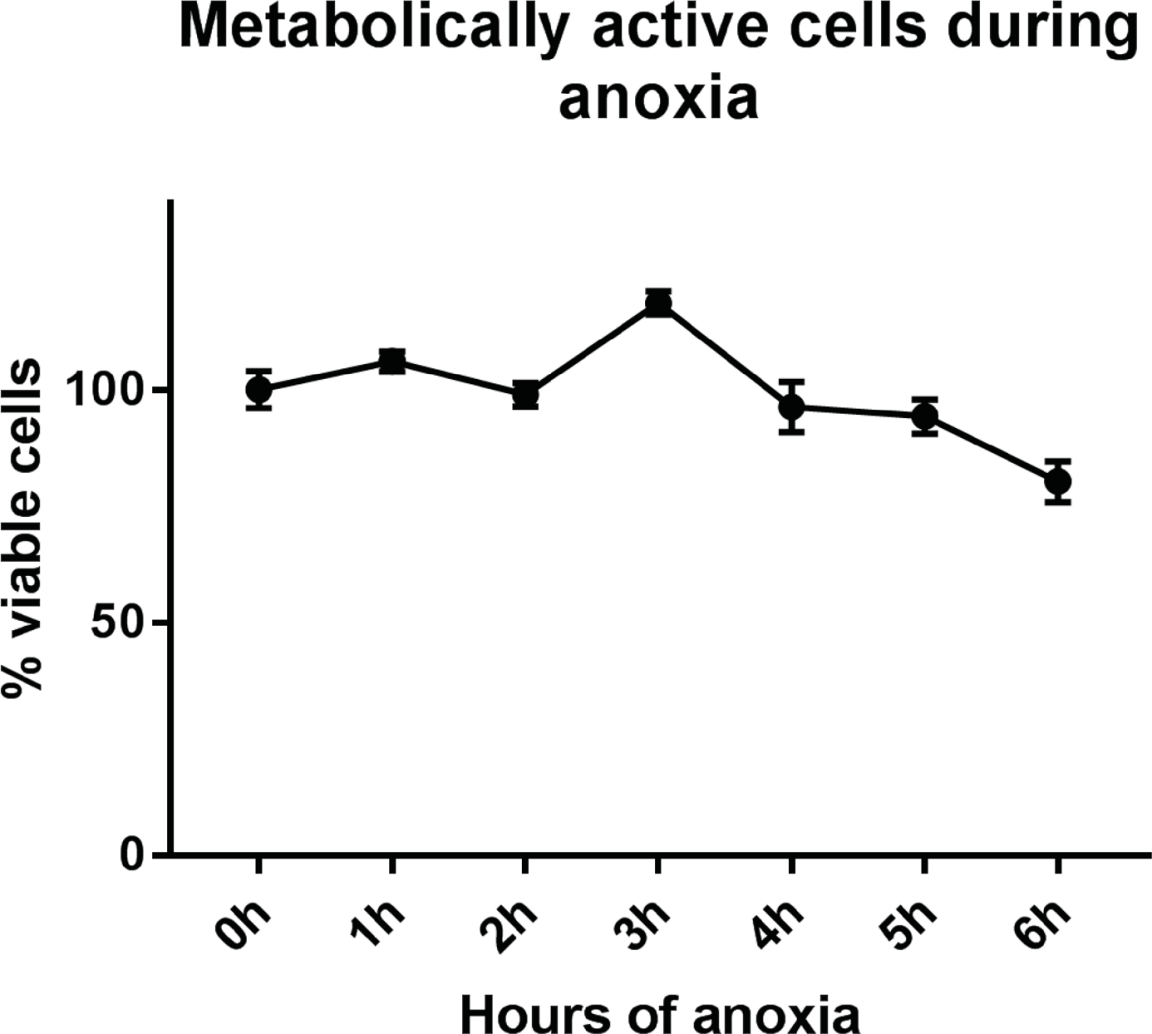
Percent viable cells after culturing under anoxic conditions. Primary muscle cells cultured in anoxic conditions triggered by oxyrase (EC-oxyrase) for 0–6 h, followed by cell viability measurements (CellTiter-Glo, Promega). The muscle cells were metabolically active during the whole period. The graph represents the average of three independent experiments (±SEM).

Apoptosis in skeletal muscle is still relatively unexplored. Skeletal muscle fibres are multinucleated and the mitochondrial content differs between fibre types, and these factors add complexity to apoptotic mechanisms in these muscle [9, 16]. Also, skeletal muscle cells can adopt to hypoxic conditions, which is unlike other cell types, see e.g. [17]. In a recent review, the authors discuss the fact that O_2_ levels actually controls myogenesis and muscle regeneration, showing that hypoxia (3–6% O_2_ levels) could in fact promote myogenesis, while anoxia (oxygen levels below 1% O_2_) appears damaging to cell differentiation [18]. To achieve anoxic conditions, an enzymatic oxygen scavenger (EC-Oxyrase) was added to differentiated muscle cells. This scavenger decreased O_2_ (S1 Fig) and after 4h it reached <0.4 mm Hg (less than 0.5% O_2_ saturation). The viability of cells (the amount of ATP measured each hour between 0 and 6 h) was not significantly changed during anoxia indicating that the muscle cells were metabolically active (Fig 1), capable of driving the apoptosis process. The presence of ATP favours and promotes apoptosis [19], and experiments in cardiac myocytes demonstrated a positive correlation between level of ATP and percentage of apoptotic cells during hypoxia [20]. Moreover, induction of apoptosis in C2C12 mouse muscle myoblasts demonstrated positive correlation between increased caspase-3 activity and sustained ATP levels [21].

**Fig 2:**
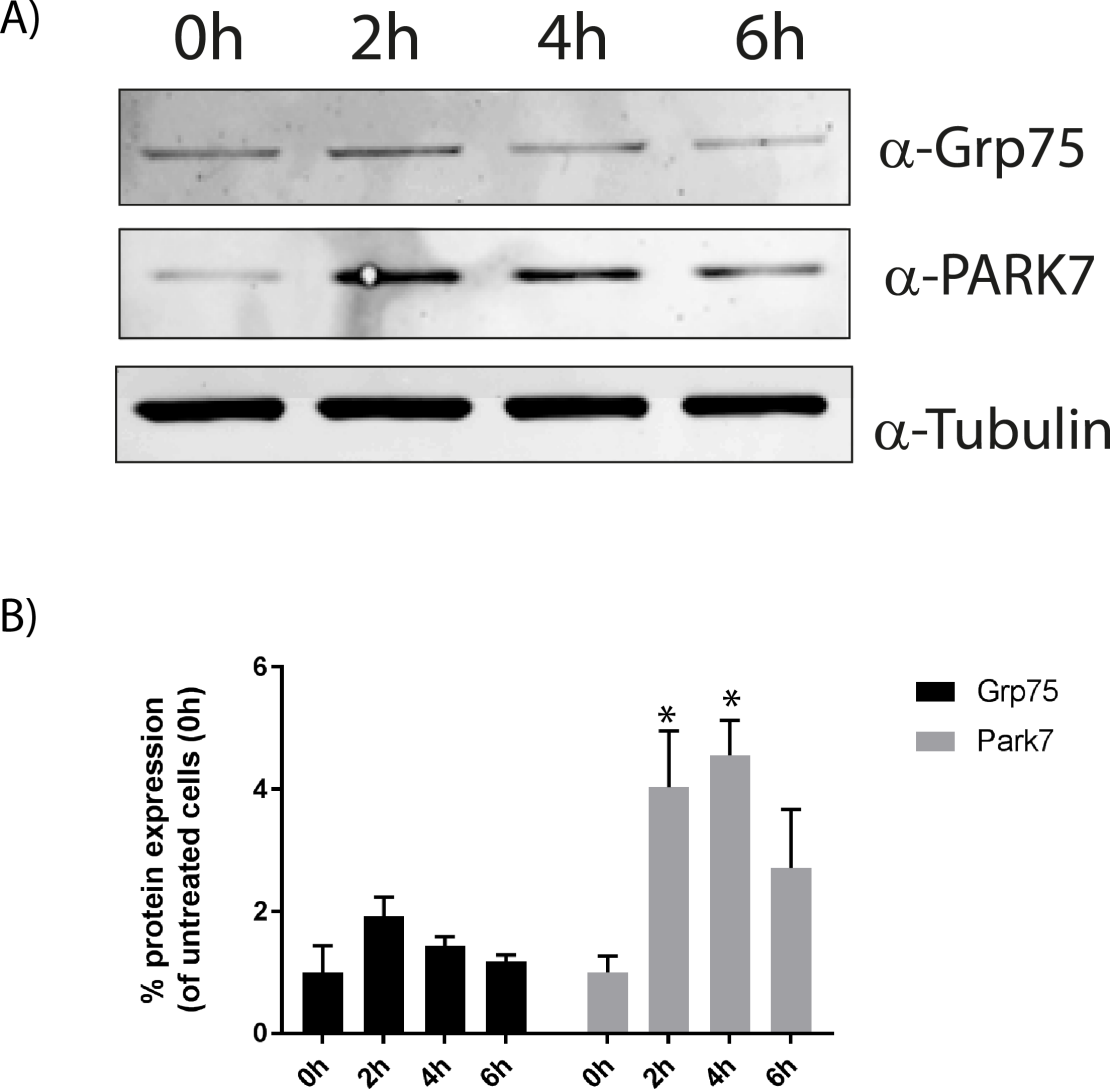
Expression of anti-apoptotic regulators. A) A representative western blot showing the transient expression level of PARK7and Grp75 during anoxia for up to 6h. Cell lysates were subjected to western blotting using antibodies to Grp75, PARK7 and tubulin (loading control). B) Expression of Grp75 and PARK7 relative to tubulin. Band intensities of each protein in the western blots, as presented in A, were quantified, and the relative band intensity of each protein was normalised to tubulin. The control cells (0h) was set to one. The graph represents the average of three independent experiments (+SEM). Asterisks denote significant differences between control and anoxic conditions (*p<0.05).

Cells exposed to stress try to suppress the apoptotic program, and try to repair the damage [22]. Grp75 (Hsp70) has previously been shown to regulate the release of cytochrome c and pro-apoptotic factors from the mitochondria [23]. PARK7 also protect against apoptosis by decreasing the expression of pro-apoptotic factors and by inhibiting caspase activation [24]. Although not significant for all time points, we observed a transient expression of both Grp75 (Hsp70) and PARK7 (Fig 2), suggesting a transient protecting mechanism. Subsequently, we performed all experiments at 6h anoxia, the time point chosen when the transient protecting mechanism of Grp75 and PARK7 was accomplished (Fig 2).

**Fig 3:**
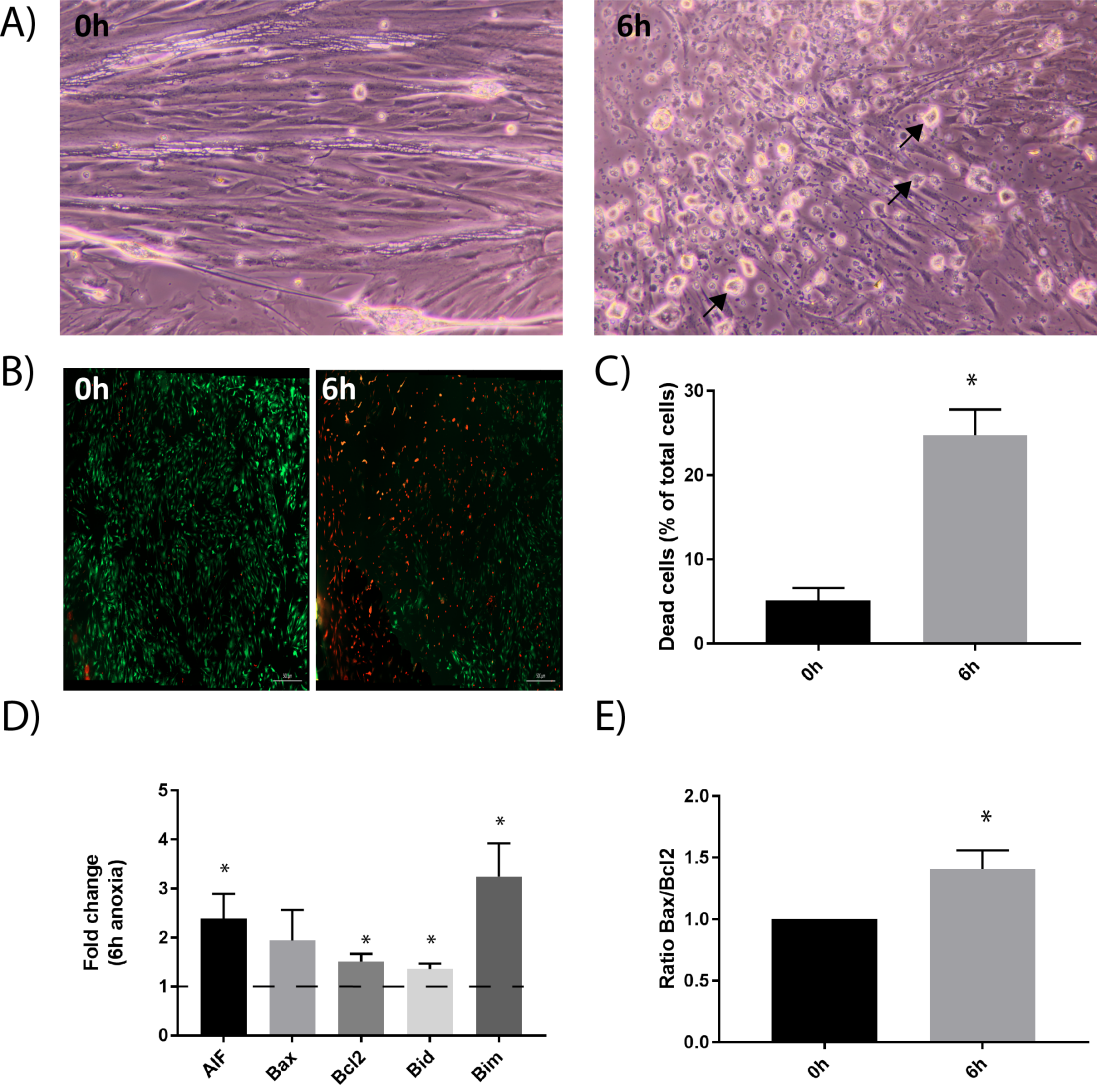
Activation of cell death upon anoxia treatment. A) Light microscopy pictures demonstrate morphological changes during 6h anoxia treatment. Arrows indicate rounded, dead cells. B) Live/dead analysis demonstrated increased number of dead cells during 6h anoxia. The method was based on the simultaneous determination of live and dead cells with two probes that recognises cell viability. Green staining (Calcein AM) demonstrated live cells, while red staining demonstrated dead cells (Ethidium homodimer). Scale bar indicated. C) Fluorescence images, as presented in B, were quantified using Image J Cell Counter plugin, to obtain percentage of dead cells during anoxia. The graph represents the average of two independent experiments, quantifying a minimum of nine pictures each (+SEM). Asterisks denote significant differences between control and anoxic conditions (*p<0.05). D) The relative mRNA expression of pro- and anti-apoptotic factors (Bax, Bcl-2, Bid, Bim and AIF) increased during 6h anoxia. Bars show the relative mRNA expression in cells subjected to anoxia compared to untreated cells (which are set to one, baseline indicated with dotted line). The data is presented as the average mean of at least six independent experiments performed in technical triplicates, (+SEM). Asterisks denote significant differences between control and anoxic conditions (*p<0.05). E) Bars show the ratio of Bax/Bcl-2 in primary muscle cells during anoxia compared to untreated cells (which are set to 1) (+SEM). Asterisk denote significant difference between 0h and 6h anoxia.

Anoxic conditions led to rounding up and dying myoblasts and some myoblasts appeared detached (Fig 3A). To confirm that actual cell death was taking place we performed a live/dead viability/cytotoxicity assay to quantify apoptotic cell death (Fig 3B and 3C). The Bcl-2 proteins are central regulators of mitochondrial permeability and release of pro-apoptotic molecules. The Bcl-family play a pivotal role in deciding whether a cell will die or live, and are useful targets upstream of caspase activation, indicating early onset of apoptosis. Bcl-2 and Bcl-xL are anti-apoptotic members localized in the mitochondrial and endoplasmic reticular membranes, as well as in the nuclear envelope. In the mitochondria, Bcl-2 and Bcl-xL preserve mitochondrial integrity and prevent the subsequent release of apoptotic molecules. Anoxic conditions led to a significant increase (P<0.05) in the mRNA expression of anti- (Bcl-2) and pro- apoptotic markers (Aif, Bid and Bim) (Fig 3D), suggesting onset of apoptosis. The ratio between Bcl-2 and Bax determines the susceptibility of the cells to undergo apoptosis. An increased Bax/Bcl-2 ratio favours cytochrome c release from mitochondria to cytoplasm, and subsequently activate the mitochondria-mediated signalling pathways [6, 25]. Our experiments demonstrate a significant increase in Bax/Bcl-2 ratio after 6h of anoxia (Fig 3E), suggestive of onset of the intrinsic pathway. Downstream of this checkpoint are two major execution programs: mitochondrial dysfunction and the caspase pathway.

**Fig 4:**
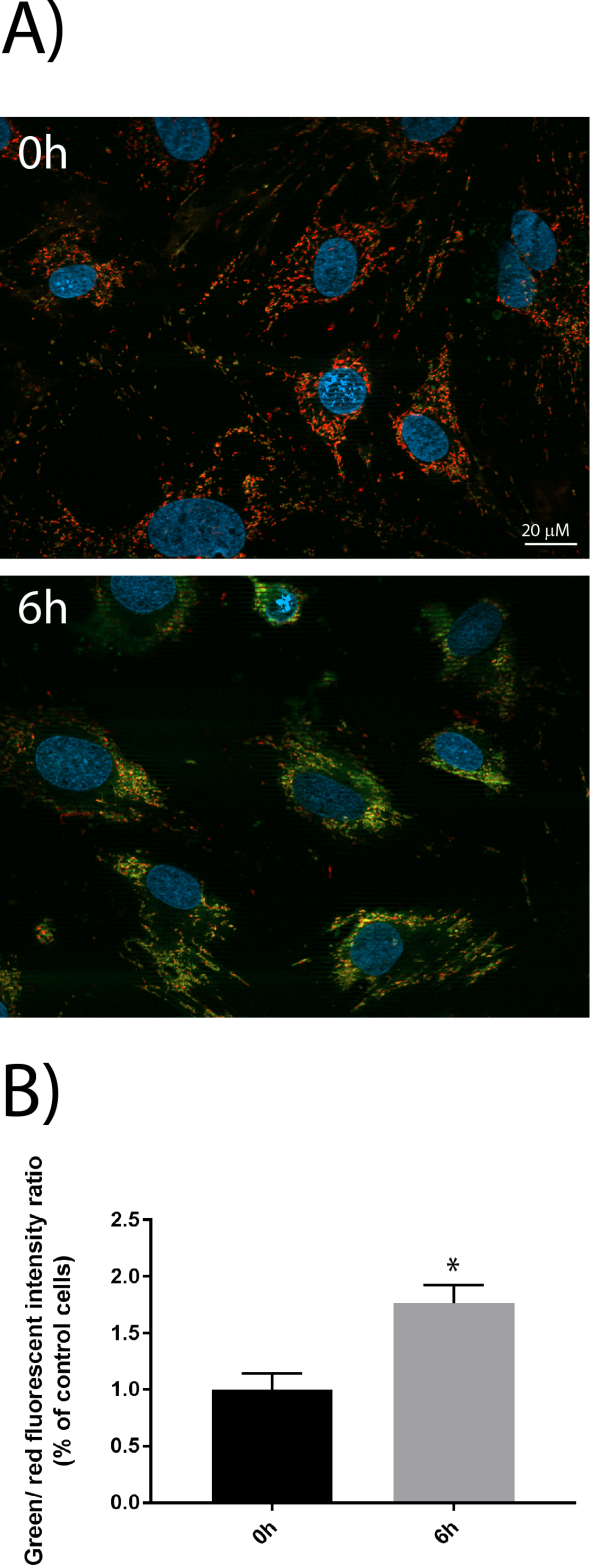
Mitochondrial membrane potential during the anoxia-culturing period. Mitochondrial membrane potential decreases during anoxia for 6h. A) Live cell fluorescence microscopy analysis of primary muscle cells treated with the apoptosis marker JC-1. This dye selectively enters the mitochondria, and changes the colour from red to green as the membrane potential decreases. Red staining demonstrates normal function of mitochondria, while green staining demonstrates loss of mitochondrial membrane potential (indicator of apoptosis). All images were captured using the same settings. Nuclei were stained with Hoechst. Scale bar 20 μM. B) Quantification of images in A using at least four randomly chosen images, demonstrating the relative intensity of green emission compared to red emission (+SEM). Asterisks denote significant differences between control and anoxic conditions (*p<0.05).

**Fig 5:**
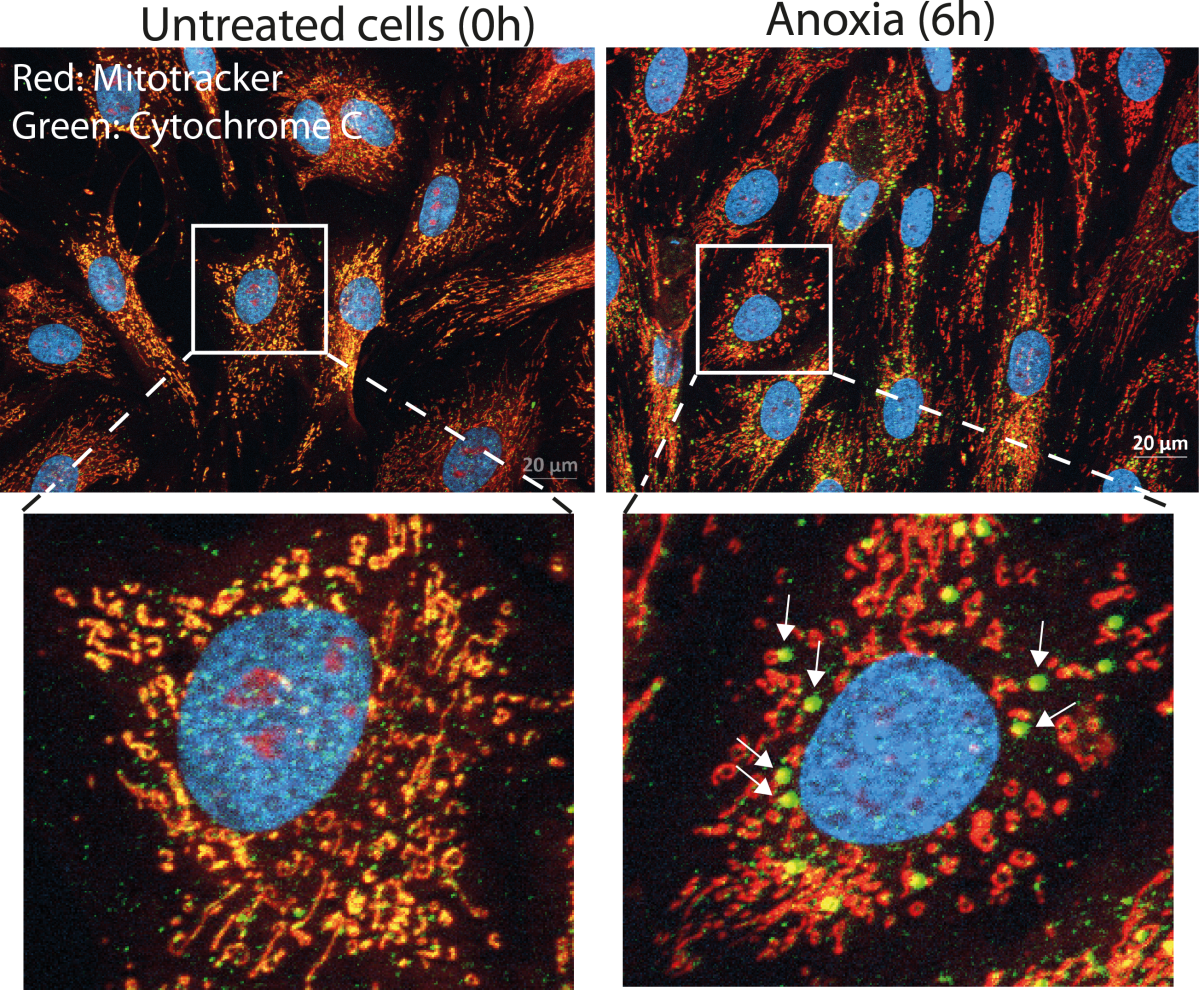
Cytochrome c localization during the anoxia-culturing period. Cytochrome c was released from mitochondria during anoxia for 6h. Differentiating cells were fixed (ice-cold ethanol) and immunostained with mouse anti-cytochrome c, followed by Alexa 488-conjugated goat anti-mouse (green) before fluorescence microscopy analysis. Mitochondria were stained using mitotracker (red), nuclei were stained with Hoechst (blue). Scale bar 20 μM. Arrows show release of cytochrome c in close proximity to the mitochondria area.

Mitochondrial dysfunction includes a change in the mitochondrial membrane potential, production of reactive oxygen species (ROS), opening of the permeability transition pore (PTP), and the release of the intermembrane space protein, cytochrome c. Released cytochrome c activates Apaf-1, which in turn activates a downstream caspase program. The experiments clearly show a decrease in membrane potential, and the relative ratio of green/red fluorescence intensity increases significantly during anoxia (Fig 4A and 4B). We also observed cytochrome c release from the mitochondria (Fig 5).

**Fig 6:**
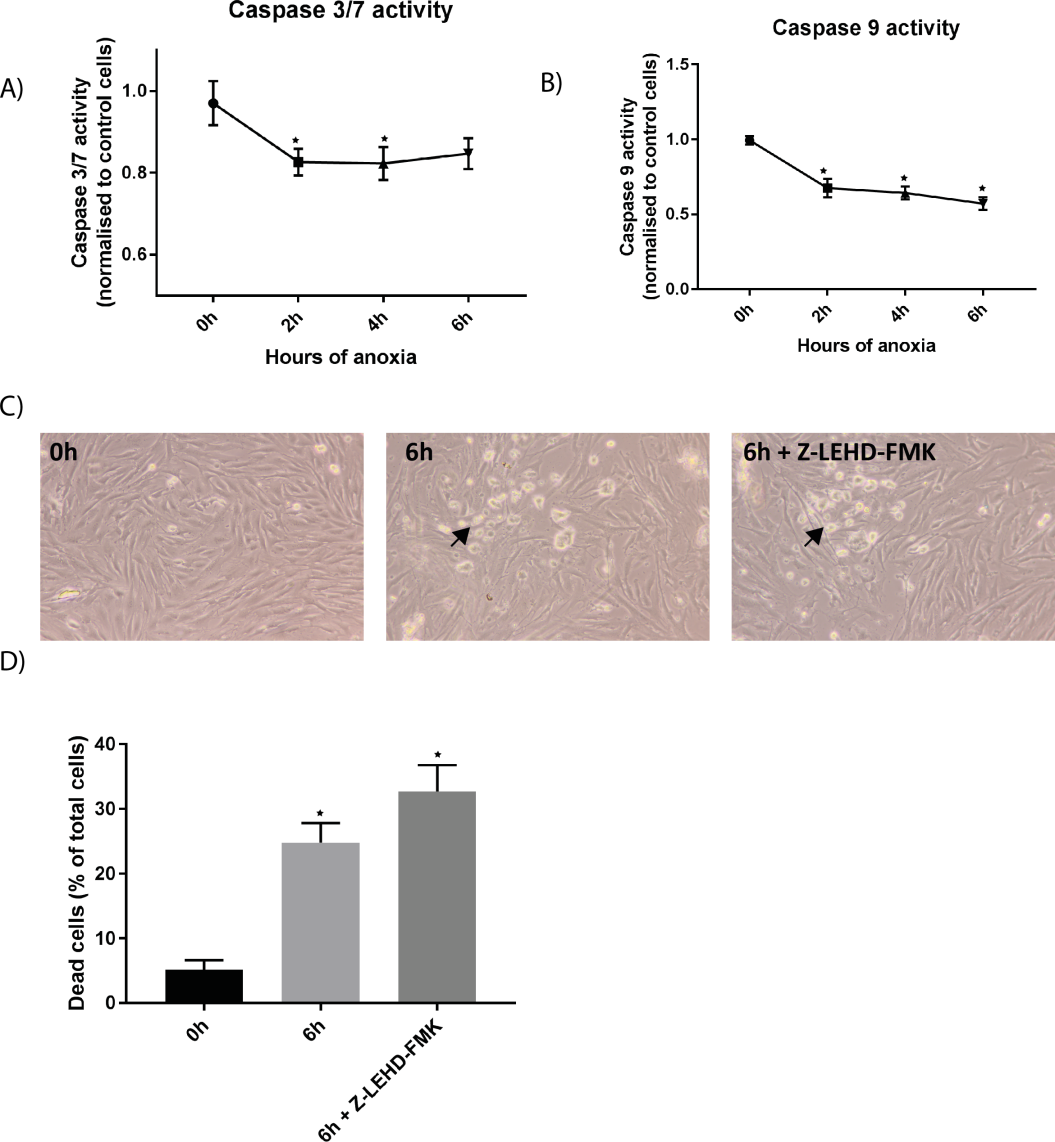
Caspase activity measured during the anoxic culturing period. The activities of caspase 3/7 A) and caspase 9 B) measured at 2h, 4h and 6h were reduced during anoxia, compared with control cells. Asterisks denote significant differences between control and anoxic conditions (*p<0.05) ± SEM. C and D) Differentiated muscle cells were pre-incubated with 20 μM Z-LEHD-FMK (irreversible caspas-9 inhibitor) for 30 min before exposure to anoxic conditions. C) Light microscopy pictures demonstrates morphological changes during anoxia treatment. Arrows indicate rounded up, dead cells. D) Live/dead analysis demonstrate increased number of dead cells during 6 h anoxia, but this is unaffected by the irreversible caspase-9 inhibitor. Fluorescence images, as presented in Fig 3, were quantified using Image J, to obtain percentage of dead cells during anoxia. The graphs represents the average of two independent experiments, quantifying a minimum of nine pictures each (+SEM). Asterisks denote significant differences between control and anoxic conditions (*p<0.05).

Caspases are a family of conserved cysteine proteases that play an essential role in apoptosis [26]. The roles of caspase 3/7 and 9 in the intrinsic pathway of apoptosis in many biological systems are well known, (see review [6]). Nevertheless, to our knowledge, there are only few examples of caspase involvement of the intrinsic pathway in skeletal muscle. Caspase 3 and 9 are shown to be involved in muscle fibre death in different muscular diseases [27], and Siu *et al*., demonstrated increased caspase-3 activity and release of cytochrome c, as well as increased AIF expression in differentiated myotubes during H_2_O_2_-mediated oxidative stress [8]. We did not observe an increase in caspase 3/7 or caspase 9 activity, (Fig 6A and 6B); on the contrary, we observed a reduced activity. There is evidence showing that cells exposed to stress can initiate a suicide program that does not rely on caspase activation [28]. The process by which myoblasts and multinucleated cells undergo apoptosis in skeletal muscle is probably quite different, and there are reports demonstrating that apoptosis in differentiated muscle cells can be performed both in caspase dependent and independent manner, and that differentiated cells are more likely to be resistant to apoptosis compared to myoblasts [8, 16, 29]. Experiments performed on C2C12 mouse muscle cells demonstrated a differential activation of caspases in myoblasts and myotubes, depending on the reagent inducing apoptosis [16]. Although a reduction in caspase activity was observed, the variability observed was high (Fig 6). It is difficult to obtain 100% myotubes in *in vitro* muscle cell systems. Characterization of the bovine muscle cell model system shows that the fusion index (i.e. percentage of total nuclei incorporated in myotubes) after 5 days in differentiation media is 50–60% [11], which is normal for cell cultures. This heterogenic cell population probably accounts for the high variability observed. Apoptosis-inducing factor (AIF) is a well-known mediator of caspase independent cell death, and findings of an increase in AIF gene expression in the present study demonstrate that AIF release promotes this process by contributing to loss of mitochondrial function [7]. The noticed increased AIF-expression and reduced caspase activity as in this study (see Figs 3A and 6, respectively) also align with a recent report [30] indicating that the AIF was important for stretch-induced apoptosis in myoblasts in a caspase-9 independent way [30]. Using the irreversible caspase 9-inhibtor Z-LEHD-FMK, we observed no inhibition of apoptosis, supporting the notion that the observed cell death in muscle cells upon anoxic conditions was independent of caspase-9 (Fig 6C and 6D). Finally, to date, ten major caspases have been identified [6], and other caspases than caspase 3/7 or 9 could be important in muscle cells during apoptosis. The caspases are also shown to play a non-apoptotic role during muscle differentiation, and there are numerous similarities between caspase-mediated apoptosis and cell differentiation [31]. Caspase 3 activation and muscle differentiation are shown to be inhibited when caspase-9 expression is reduced. Interestingly, the same effect is observed when Bcl-Xl is overexpressed, suggesting that both these genes are important for normal muscle differentiation [32, 33]. Thus, two genes that normally regulate and/or control commitment to apoptosis are in fact used for very different purposes during differentiation. The exact role of caspases post-mortem remains to be examined further. Huang et al demonstrated that caspase 3 was activated in the early post-mortem (peaking at 0.5 days post-mortem) period in muscle samples from *Longissimus thoracis*, but this activation was transient and only effective during the early post-mortem period [5]. Caspases are expressed only transiently, and apoptotic cells can die and disappear rather quickly. The time from initiation of apoptosis to completion can occur as quickly as 2–3 hours. Therefore a false negative can occur if the assay is done too soon or too late [6], and as such we cannot rule out the importance of caspases in our bovine muscle cells during anoxia.

**Fig 7:**
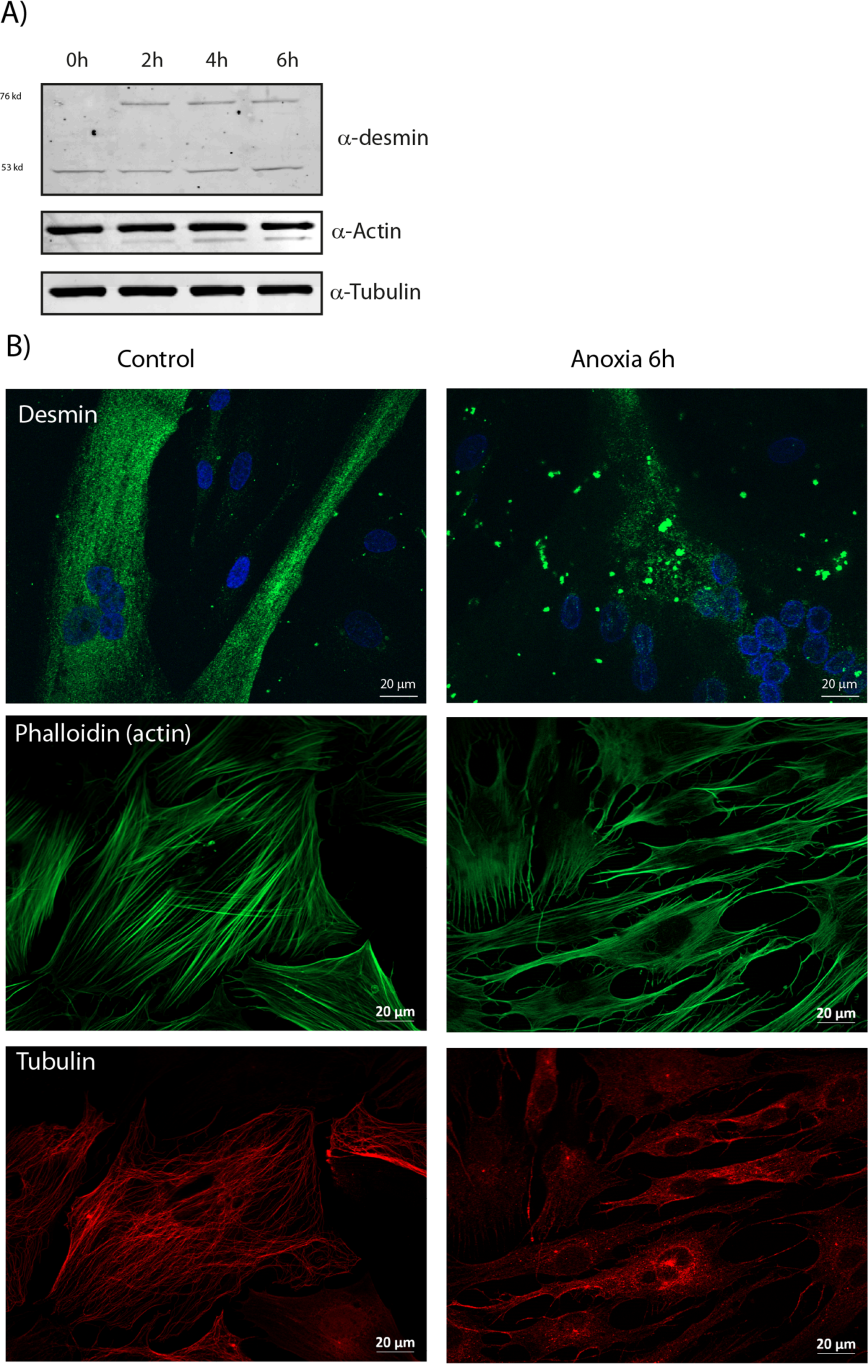
Cytoskeletal changes during anoxia. A) A representative western blot showing formation of desmin aggregates, and degradation of actin during anoxia (0-6h). The Tubulin protein band was unchanged. Cell lysates were subjected to western blotting using antibodies to tubulin, actin and desmin. B) Anoxia induces cytoskeletal re-organization. Differentiating cells (fixed with 2% PFA) and immunostained with rabbit anti-Desmin and mouse anti-α-tubulin, followed by Alexa 488-conjugatede goat anti-rabbit (green) and Alexa 546-conjugated goat anti-mouse (red) before fluorescence microscopy analysis. Actin was stained using Alexa-488 Phalloidin; nuclei were stained with Hoechst (blue). A reorganization of the cytoskeleton was clearly visible with both desmin, tubulin and actin staining. Scale bar 20 μM.

Breakdown of the cytoskeleton is an indicator of apoptosis. Actin and desmin are known substrates for proteolytic enzymes, and microtubules are known to disassemble during early apoptosis. In apoptotic cells, actin is cleaved into smaller fragments, and this affect cellular morphology. In order to study the effect on cytoskeleton during anoxia, we measured protein abundance and performed immunofluorescence of actin, tubulin and desmin,. Our experiments demonstrate degradation of actin, (Fig 7A), and this affected the actin cytoskeleton (7B). Although we observed depolymerisation of tubulin during anoxia (Fig 7B), we did not observe degradation of tubulin, and total protein amount of tubulin was unaffected during anoxia (Fig 7A). Desmin has also been implicated as a hallmark of the apoptotic pathway because it is cleaved specifically at the 263 aspartic acid residue, by caspase-6 *in vitro*. It is also cleaved in myogenic cells after treatment with Tumour necrosis factor-α (TNF-α) [34]. TNF-α is a major mediator of inflammation, involved in apoptosis, and previous experiments have shown that TNF-α overexpression induces cytoplasmic desmin aggregation in cardiomyocytes [35]. Consistent with these observations, desmin formed large aggregates during anoxia (Fig 7A and 7B) in our experiments.

Altogether, these results suggest that muscles will enter apoptosis after few hours when available oxygen in the muscle cells diminishes post-mortem. The muscle cells try to protect themselves from the harmful environment post-anoxia by expressing protective markers such as Grp75 and PARK7. Anoxic conditions led to an increased expression of pro- and anti-apoptotic markers, a loss of mitochondrial membrane potential, release of cytochrome c, and finally a re-organization of cytoskeletal proteins, degradation of actin and aggregation of desmin. These are all hallmarks of the intrinsic pathway, especially the release of cytochrome c from mitochondria, which is essential for intrinsic apoptosis. Increased Ca^2+^ accumulation is a trigger for the release of cytochrome c from the mitochondrial intermembrane space into the cytosol where it can activate caspases and lead to apoptosis.

## Supporting information

S1 Fig Saturation of O_2_. Saturation of O_2_ in the growth medium was measured by an oxygen probe in cell culture plates with or without addition of Oxyrase. The data is presented as mean +/- SD from three independent experiments.
(TIF)
