# Supplementary figures and images for: Primary bovine skeletal muscle cells enters apoptosis rapidly via the intrinsic pathway when available oxygen is removed
Source: PLoS One. 2017 Aug 8;12(8):e0182928. doi: 10.1371/journal.pone.0182928 (PMC5549745; doi:10.1371/journal.pone.0182928)

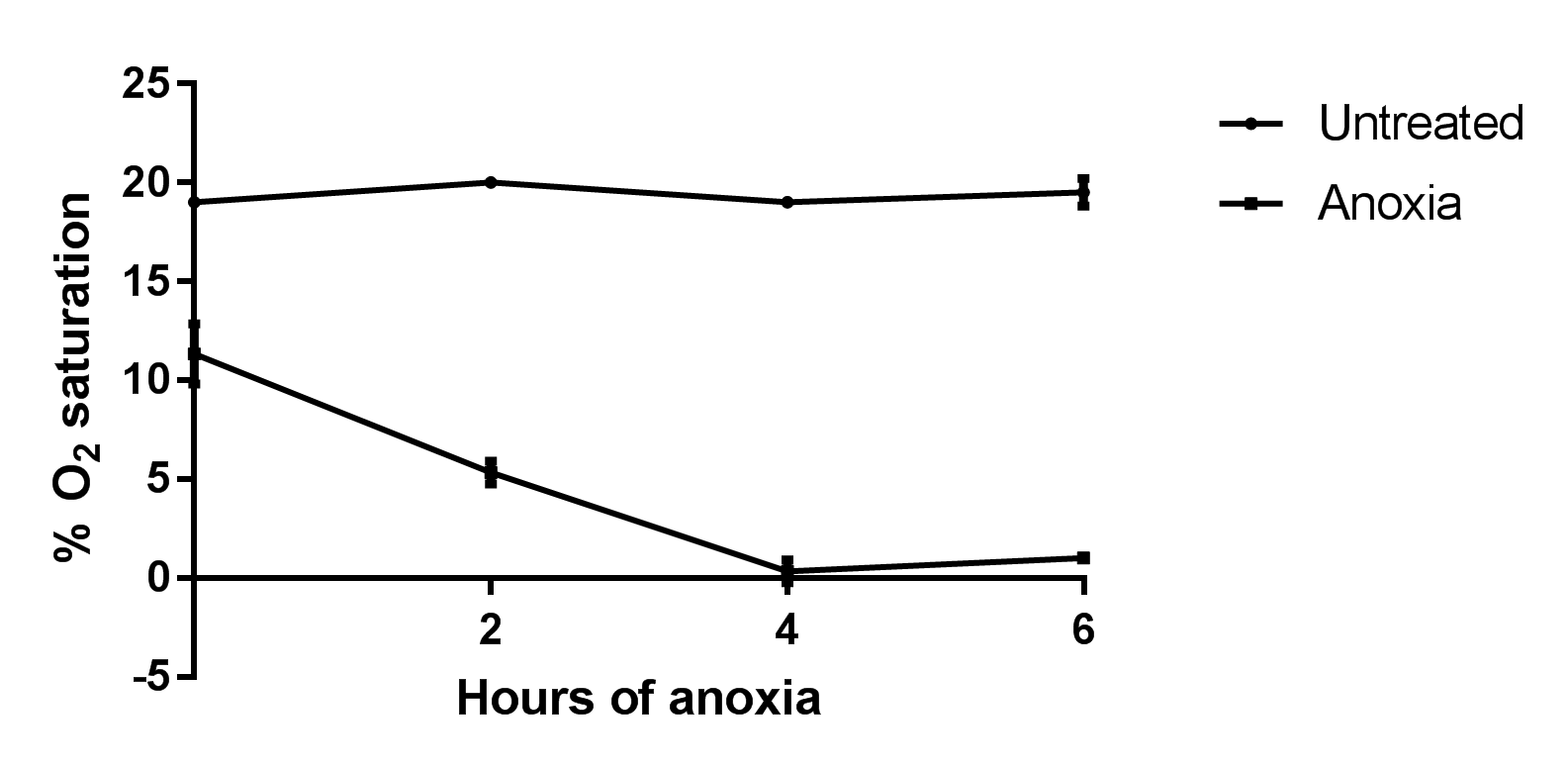

Supplement: S1 Fig — Saturation of O2 in the growth medium was measured by an oxygen probe in cell culture plates with or without addition of Oxyrase. The data is presented as mean +/- SD from three independent experiments. (TIF) [file pone.0182928.s001.tif]
